# Autistic Traits Are Associated with Suicidal Thoughts and Behaviors Among Patients with Borderline Personality Disorder

**DOI:** 10.3390/brainsci15040340

**Published:** 2025-03-25

**Authors:** Barbara Carpita, Benedetta Nardi, Federico Giovannoni, Chiara De Felice, Federica Tranchese, Chiara Bonelli, Gabriele Massimetti, Ivan Mirko Cremone, Stefano Pini, Maria Rosaria Anna Muscatello, Liliana Dell’Osso

**Affiliations:** 1 Department of Clinical and Experimental Medicine, Section of Psychiatry, University of Pisa, 67 Via Roma, 56126 Pisa, Italy; barbara.carpita@unipi.it (B.C.); f.giovannoni10@gmail.com (F.G.); c-defelice@hotmail.com (C.D.F.); f.tranchese@studenti.unipi.it (F.T.); chiarabonelli.95@hotmail.it (C.B.); gabriele.massimetti@unipi.it (G.M.); ivan.cremone@gmail.com (I.M.C.); stefano.pini@unipi.it (S.P.); liliana.dellosso@gmail.com (L.D.); 2 Department of Neurosciences, School of Specialization in Psychiatry, University of Messina, 98122 Messina, Italy; maria.muscatello@unime.it

**Keywords:** borderline personality disorder, autistic traits, suicidality, suicidal behavior, autism spectrum, personality disorder

## Abstract

**Background:** Borderline personality disorder (BPD) is a pervasive mental health condition characterized by a heightened risk of suicidal behavior. Emerging research has suggested a potential overlap between BPD and subthreshold autistic traits (ATs), raising the possibility that these traits may influence the development, course, and severity of BPD, particularly in relation to suicidal ideation and behaviors. This study aims to evaluate the relationship between suicidal ideation, suicidal behaviors, and ATs in individuals with BPD. **Methods:** We assessed 106 subjects with BPD using the mood spectrum self-report version (MOODS-SR) of the Adult Autism Subthreshold Spectrum (AdAS Spectrum) questionnaire. The sample was divided into three groups based on suicidal ideation and behaviors. Non-parametric tests compared AdAS Spectrum scores, while Spearman’s correlation assessed the relationships between AdAS Spectrum scores and suicidality. Logistic regression analyses were conducted to identify predictive AdAS Spectrum domains for suicidal ideation and behaviors. **Results:** Subjects with suicidal behaviors and suicidal ideation showed significantly more autistic features than non-suicidal subjects. Correlation analysis revealed that all AdAS Spectrum domains, except empathy, were significantly correlated with both suicidal ideation and behaviors, with stronger correlations for suicidal behaviors. Moreover, restricted interests, rumination, and sensory sensitivity emerged as significant predictors of suicidal ideation, while the lack of empathy was a significant predictor of suicidal behavior. **Conclusions:** Our results confirm a strong correlation between the presence of ATs and suicidality in subjects with BPD, in particular highlighting rumination, altered sensitivity, and empathic deficits as specific predictors of suicidal thoughts and behaviors.

## 1. Introduction

Borderline personality disorder (BPD) is a mental health condition marked by a persistent pattern of unstable relationships, fluctuating mood, and a distorted self-image, typically emerging in early adulthood. Key features of BPD include a deep fear of abandonment, volatile relationships that swing between idealization and devaluation, instability in self-worth, impulsive behaviors, self-harm, intense emotional reactivity, chronic feelings of emptiness, and occasional paranoid thoughts or dissociation [1]. The lifetime prevalence of BPD is estimated to be about 5.3%, with women making up roughly 80% of those affected [2,3]. Personality disorders, including BPD, are commonly encountered in clinical settings, with about 45% of outpatient cases presenting with personality disorders alongside other conditions. BPD, in particular, has received extensive research attention due to the treatment challenges it presents. Impulsivity and self-injurious tendencies also make BPD patients more susceptible to suicidal behavior, with studies showing that individuals with BPD attempt suicide an average of three times over their lifetime, and up to 10% die by suicide [4,5,6]. Because of its early onset, high prevalence, and associated suicide risk, BPD represents a significant public health concern, necessitating specialized rehabilitation programs and ongoing monitoring.

Recently, attention has shifted toward understanding how the presence of autistic traits (ATs) might intersect with BPD, influencing the disorder’s development and course. A growing body of research has explored how subthreshold ATs—traits that are less pronounced than those seen in autism spectrum disorder (ASD)—may affect individuals with BPD [7,8,9,10,11]. The interest in subthreshold ATs has surged due to increasing evidence suggesting that these traits may not only heighten vulnerability to developing other psychiatric disorders but also influence the trajectory of existing conditions [12,13,14]. Initially, research focused on first-degree relatives of individuals with autism spectrum disorder (ASD), who displayed personality characteristics resembling those of people with ASD, though less pronounced. These traits included inflexibility, social withdrawal, a detached personality, and narrowly focused interests [15,16]. Later studies examined ATs in both clinical and non-clinical populations, discovering that individuals with higher levels of ATs were more likely to suffer from other mental health disorders [17,18,19,20,21,22]. Subthreshold ATs have been found to significantly impact quality of life and contribute to the development of additional mental health challenges, including suicidal ideation and behaviors.

Building on these findings, recent clinical observations have suggested a potential link between BPD and the spectrum of autism [23]. Beyond the simple question of co-occurrence, some researchers have proposed that there may be shared underlying factors contributing to both conditions. Several studies have highlighted a significant overlap between ATs and BPD, suggesting that specific traits associated with autism—such as difficulties in communication, empathy, and mentalization—could play a role in the development of BPD, thereby increasing susceptibility to the disorder [11]. Recent research has also explored the impact of ATs on particular BPD symptoms, helping to clarify how these traits might influence the disorder’s progression [21].

In a similar vein, both BPD and ASD, as well as ATs, have long been correlated with an increased risk of suicidal thoughts and behaviors. Suicide mortality rates are generally higher in males, while females tend to experience more frequent suicidal thoughts and behaviors [24,25,26]. In order to reduce suicide rates, it is crucial to identify specific risk factors, not only within patient populations but also across the general public. Although the path to suicide is multifaceted, recent studies emphasize the significant role psychological factors play in this process [27]. In this framework, many researchers have suggested that a variety of psychological features associated with autism spectrum, such as perfectionism, neuroticism, and introversion, are strongly correlated with greater suicidal tendencies [28,29,30]. Moreover, an expanding body of research indicates that individuals with autism spectrum are at a heightened risk of suicidal thoughts, planning, attempts and, tragically, suicide [31,32,33,34,35,36]. For example, a recent study highlighted how ATs, and specifically communication and imagination deficits, were more represented in subjects with a positive history of multiple suicide attempts and even how undiagnosed ASD is correlated to suicidal behavior [37,38]. Similarly, prisoners with clinically relevant ATs have been reported to be more likely to have thoughts about self-harm and suicide, and to have attempted suicide during their lifetime [39]. In light of this evidence, a more effective way to identify suicide risk could involve screening for ATs in adults with mental health conditions, as well as screening for suicidal tendencies in individuals on the autism spectrum.

Within this context, the aim of our study was to evaluate the relationship among suicidal ideation, suicidal behaviors, and autism spectrum in a sample of subjects with BPD, specifically focusing on identifying specific autism spectrum symptoms predictive of suicidal ideation and/or suicidal behaviors. The primary hypothesis of our study was that higher levels of autistic traits would be linked to increased suicidality, with specific autistic dimensions correlating with heightened suicidal ideation and behaviors. We anticipated that these associations would differ between individuals with only suicidal ideation and those displaying both ideation and behaviors. We also hypothesized that certain characteristics, as measured by the AdAS Spectrum, would vary across three groups: non-suicidal, suicidal ideation only, and suicidal behaviors. Furthermore, we proposed that these domains could not only help predict the presence of suicidal behaviors but also provide insight into the progression from suicidal ideation to more severe actions.

## 2. Materials and Methods

### 2.1. Study Sample and Procedures

For this study, we recruited a consecutive sample of 106 subjects with a clinical diagnosis of BPD attending the psychiatric clinic of the Azienda Ospedaliero Universitaria Pisana. All participants were recruited between November 2021 and January 2022. Subjects with mental disability, poor collaboration skills, an age under 18, language or intellectual impairment that would make it difficult to complete the exams and continuous psychotic symptoms at the moment of evaluation were excluded from the recruitment procedures. All enrolled participants were assessed by experienced psychiatrists to determine the presence or absence of mental disorders. BPD diagnoses were made in accordance with the DSM-5 criteria by specialists in the field. All participants were evaluated individually during a routine specialist visit. The Declaration of Helsinki was followed in the conduct of the study. All hiring and evaluation practices were approved by the Azienda Ospedaliero-Universitaria of Pisa Ethics Committee. All participants provided written informed consent following a thorough explanation of the study and a chance for questions. In compliance with Italian law, subjects received no compensation for their participation.

All participants were evaluated with the mood spectrum self-report (MOODS-SR) questionnaire in order to assess the presence of suicidal ideation or suicidal behaviors, and Adult Autism Subthreshold Spectrum (AdAS Spectrum) characteristics.

### 2.2. Measures

#### 2.2.1. The MOODS-SR

The MOODS-SR is a questionnaire designed to assess temperamental features and a wide spectrum of mood symptoms throughout life. It is made up of 7 domains with 160 items in total. The Cronbach’s alphas for the Italian version varied from 0.72 to 0.92. In earlier research, the MOODS-SR was used to assess suicidality (suicidal ideas and behaviors), which was determined by questions 102 through 107 [40,41,42]. In particular, items (102) “You thought that life was not worth living?”; (103) “You hoped that you would not wake up in the morning, or that you would die in an accident or from something like a heart attack or a stroke?”; (104) “You wanted to die or hurt yourself?”; and (105) “If you felt that you wanted to die, did you have a specific plan to hurt or kill yourself?”; were used to assess the presence of suicidal ideation. Items (106) “Did you actually try to kill yourself?” and (107) “If you tried to kill yourself, did you require medical attention?” were used to assess suicidal behaviors, as in previous studies [41,42,43,44].

#### 2.2.2. The AdAS Spectrum

The AdAS Spectrum is a 160-item self-report tool that is designed to assess the broad spectrum of manifestations of full-blown ASD and subthreshold autistic traits in adults who do not have cognitive or linguistic deficits. The questionnaire was developed to include in the assessment both attenuated and atypical symptoms, as well as personality traits and behavioral manifestations that may be associated with ASD but may also be present in subthreshold forms. It is made up of 7 domains that look into symptoms associated with childhood and adolescence, verbal and nonverbal communication deficiencies, empathy alterations, a propensity towards rigidity and routine adherence, the presence of restricted interests and rumination, and hyper- and hyporeactivity to sensory input. The validation study found strong internal consistency, excellent test–retest reliability, and convergent validity with other dimensional measures of autism spectrum [45].

### 2.3. Statistical Analysis

First, the sample was divided in three groups (lifetime non-suicidal subjects, subjects with only suicidal ideation lifetime, subjects with both suicidal ideation and suicidal behaviors lifetime) based on the responses given in the items 102 through 107 of the MOODS-SR. Specifically, subjects who answered positively to at least one item from 102 to 104, but negatively to items 106 to 107, were classified into the suicidal ideation group. In contrast, subjects who answered positively to at least one item from 106 to 107 were placed in the suicidal behaviors group, regardless of their responses to items 102 to 105.

For the purposes of this study, non-parametric tests were used since the sample did not meet the tests of normality and homoscedasticity of variance.

Chi-square and Kruskall–Wallis tests were then used to compare sociodemographic factors between groups. The Kruskall–Wallis test was used to compare the AdAS Spectrum scores between the three groups. Spearman’s correlation coefficient was employed to assess the pattern of correlations between the scores recorded on AdAS Spectrum, suicidal ideation, and suicidal behaviors.

Afterwards, a logistic regression analysis was performed in the whole sample, using the suicidal ideation as a dependent variable and AdAS Spectrum domains as independent variables to evaluate which AdAS Spectrum domains were statistically predictive of suicidal ideation.

A further logistic regression was carried out in subjects with suicidal ideation only and subjects with suicidal behaviors, using the same dependent and independent variables, to determine which AdAS Spectrum domains were statistically predictive of suicidal behaviors.

All statistical analyses were performed with SPSS version 26.0.

## 3. Results

The total sample was made up of 106 subjects with a clinical diagnosis of BPD (mean age: 36.8 ± 14.12 years). The sample was divided in three groups: 25 (23.6%) subjects without suicidal ideation or suicidal behaviors, 17 (16.0%) subjects with suicidal ideation only, and 64 (60.4%) subjects with both suicidal ideation and suicidal behaviors. The groups did not significantly differ for gender (X^2^ = 3.742; *p* = 0.154) nor for age (H = 4.571; *p* = 0.102) (see Table 1).

The Kruskall–Wallis test reported in Table 2 shows the comparison between groups of AdAS Spectrum scores.

Subjects with suicidal behaviors scored significantly higher than non-suicidal subjects in the childhood and adolescence, verbal communication, and inflexibility and adherence to routine domains. No significant differences emerged from the scores obtained in such domains among subjects with suicidal ideation and the other two groups.

Both subjects with suicidal behaviors and subjects with only suicidal ideation scored significantly higher than non-suicidal subjects in the non-verbal communication, restricted interests and rumination, and hyper–hyporeactivity to sensory stimuli domains as well as in AdAS Spectrum total score. No difference in these domain scores were found between subjects with suicidal behaviors and those with suicidal ideation only.

Subjects with suicidal behaviors scored significantly higher than subjects with suicidal ideation in the empathy domain; no significant differences emerged in the scores obtained in such domains among non-suicidal subjects and the other two groups.

Results of the correlation analysis reported in Table 3 highlight that all AdAS Spectrum domains and total scores were significantly correlated with both suicidal ideation and suicidal behaviors, with the only exception being the empathy domain, which although correlated with suicidal behaviors, was not correlated with suicidal ideation. Correlation between AdAS Spectrum domains and total score and suicidal behaviors were stronger than those with suicidal ideation with the exception of non-verbal communication and restricted interests and rumination domains.

From the results of a first logistic regression analysis carried out on the whole sample that used suicidal ideation as a dependent variable and AdAS Spectrum domains as independent variables, it emerged that the AdAS Spectrum domains of restricted interests and rumination and hyper–hypo-sensitivity to sensory input were significant positive predictors of the presence of suicidal ideation (see Table 4).

Results from a further logistic regression analysis, carried out on subjects with suicidal ideation only and suicidal ideation and behaviors, excluding non-suicidal subjects, with the same dependent and independent variables as the previous analysis, highlighted that the AdAS Spectrum empathy domain, which measures the lack of empathy or its alterations, was a significant positive predictor of suicidal behavior (see Table 5).

## 4. Discussion

The aim of our study was to evaluate the correlation between ATs and suicidal thoughts or behaviors in a sample of 106 subjects affected by BPD.

Our results highlighted a significantly higher prevalence of ATs in subjects with either suicidal ideation or suicidal behaviors compared to non-suicidal patients. These findings support the already present evidence of a greater suicidal risk in subjects with high ATs. Indeed, while suicidality has long been studied in ASD patients with results that agree on a significant increase in the risk of suicide [31,32,33,34,35,36,37,38,46,47], a growing number of studies are also suggesting a central role for subthreshold ATs as risk factors for suicidality in both clinical and non-clinical populations [47,48,49,50]. The longstanding debate over the connection between ATs and suicidal tendencies has led to the identification of several factors that may contribute to this link. For example, psychological characteristics commonly found in individuals on the autism spectrum, such as perfectionism, neuroticism, and introversion, have been strongly associated with heightened suicidal risk [28,29,30]. ATs are also linked to social difficulties, including isolation, bullying, and the need to camouflage traits to fit in [50,51,52,53]. While camouflaging may aid in social integration, it often leads to mental exhaustion, poor psychological well-being, and a sense of social disconnection, all of which contribute to suicidal behavior [54,55,56,57,58,59,60]. Additionally, cognitive inflexibility and repetitive thinking are linked to depressive and suicidal thought patterns, while ATs also increase the likelihood of developing anxiety and mood disorders, which are significant suicide risk factors [24,61,62]. Ultimately, recent research has emphasized that ATs serve as independent risk factors for suicide attempts, regardless of any comorbid conditions that may be present [37,63]. This is a crucial point, as individuals with disorders like BPD tend to exhibit higher levels of ATs compared to the general population [23]. Furthermore, studies have shown that individuals with both elevated ATs and BPD face a particularly elevated risk of suicide [64]. Collectively, these findings suggest that ATs may contribute to suicidal behavior beyond the well-established risk factors typically associated with these conditions.

Supporting this hypothesis of an association between ATs and suicidality, our results also highlighted strong correlations between autistic dimensions evaluated by the AdAS Spectrum questionnaire and the six items from the MOOD-SR questionnaire indicating suicidal ideation and/or suicidal behavior. For instance, higher scores in the childhood/adolescence domain, which evaluates early autistic manifestations, may be associated with a higher risk of developing psychiatric disorders and suicidality later in life [52,61,65]. Moreover, the domain also investigates the presence of specific problems and adversities associated with social difficulties experienced during the early years of life. Early social challenges, such as bullying, and difficulties in recognizing, processing, and externalizing traumatic experiences increase the vulnerability to develop complex post-traumatic stress disorder (cPTSD), which is itself a suicide risk factor [66,67,68].

Additionally, in line with previous evidences, difficulties in verbal and non-verbal communication, both common in ASD, were found to correlate with increased suicidality [7,40,42,69,70]. These communication challenges often lead to social anxiety, maladaptive coping strategies, and social isolation, further exacerbating the risk of suicidal thoughts and behaviors [69,70,71,72,73]. This is particularly notable in women, who may have an under-recognized ASD due to gender differences in symptom expression. In this sense, it is also interesting to note how social anxiety and BPD are more common in women and share with ASD an alteration of the ‘social brain’: according to some authors, these diagnoses could sometimes mask an unrecognized autistic spectrum in women [21,74,75].

The correlation between suicidality and the autistic traits of inflexibility and adherence to routine, although present in a lesser extent than others, which are among the weakest correlations, can be explained by a greater difficulty in adapting to changes and vital events, which could imply a greater development of post-traumatic spectrum symptoms and consequent greater suicidal risk [66,67]. On the other hand, according to other studies, the presence of traits of adherence to a routine could be a protective factor towards suicidal tendencies, possibly counterbalancing the high tendency towards loss of control and impulsive behaviors among individuals with BPD [76,77,78].

Lastly, hyper–hyporeactivity to sensory stimuli and restricted interests and rumination domains were shown not only to be correlated with suicidal ideation and suicidal behaviors but also to be significant predictors of the presence of suicidal ideation, thus configuring themselves as the domains of the autistic spectrum most associated with this dimension. Regarding the hyper–hyporeactivity to sensory stimuli domain, it is now known that the altered response to stimuli is often associated with emotional dysregulation in subjects on the autistic spectrum [77], and is associated with self-harming behaviors [79,80,81,82]. According to some recent studies, altered sensory sensitivity would not only be more present in patients with BPD [83], but can also be a predictive factor of suicidal tendencies in such a population [7,40]. Indeed, in subjects who by definition have marked impulsivity, sensory overstimulation could lead to self-injurious behaviors that serve as a coping mechanism for controlling emotional states and reducing overstimulation [82,84]. Sensory impairment could also be related to the post-traumatic hyperarousal frequently found in individuals with BPD, due to their traumatic past experiences, towards which ATs may represent a vulnerability factor [85,86,87]. Ultimately, in patients with BPD, the altered sensitivity to sensory stimuli could increase the vividness of traumatic memories, enhancing the phenomenon of post-traumatic re-experiencing and promoting the structuring of post-traumatic maladaptive symptoms and therefore also increasing the risk of suicidal thoughts or behaviors [8]. Rumination, on the other hand, may be viewed as a trans-nosographic dimension present in a variety of psychiatric disorders, including BPD, and is usually associated with a poorer prognosis [70], potentially contributing to the emergence of suicidal thoughts and actions [88,89]. These data supplement earlier research by Pelton and Cassidy [47], who highlighted that the lack of a sense of belonging and the perception of being a burden to others acted as mediating factors between ATs and suicidality.

Similarly, the empathy domain appeared to be correlated exclusively with suicidal behaviors and to be a significant positive predictor of the presence of suicidal behaviors. We know from the literature that patients with BPD are characterized by an altered emotional empathy and an altered theory of mind, contributing to misunderstandings and inappropriate social behaviors [90]. Social challenges related to a poor theory of mind may cause feelings of loneliness and isolation, which are risk factors for suicidal ideation. Additionally, deficits in theory of mind can result in negative social interactions, exacerbating isolation. Moreover, the construct of perceived burdensomeness may bear significant relevance to a particular sort of theory of mind error known as ‘over-mentalizing’. Identifying oneself as a burden to others necessitates the application of theory of mind skills, but occasionally, a mistake in evaluating another person’s mentalization can result in the inference of unduly negative and intense thoughts and feelings about the other person’s mental state, as well as the perception of oneself as a burden to others. An excessive but inappropriate mentalization has been observed in people with a variety of mental illnesses, including BPD [91,92,93]. In this framework, some authors suggested that over-mentalization can lead the subjects to think of themselves as a burden to others and that this inaccurate perception of burdensomeness may in turn ultimately represent a specific interpersonal risk factor for suicide desire and behaviors [94]. It should also be noted that a difficulty in recognizing emotions may specifically predispose individuals to engaging in self-harm behaviors without having a clear understanding of consequences for oneself and others, including the impact of one’s own death [95,96,97,98].

Our results should be seen in light of some limitations. For instance, the cross-sectional model of our research prevents us from drawing conclusions regarding both temporal and causal connection between the variables. Second, considering that we used self-report questionnaires, participant over- or underestimation of symptoms could potentially skew the outcomes of the scores. Finally, the limited size of our sample limits how broadly the findings may be applied.

## 5. Conclusions

Our results confirm a strong correlation between the presence of ATs and suicidality in subjects with BPD, in particular highlighting rumination and altered response to sensory stimuli as predictors of suicidal ideation, while empathic deficits would be specific predictors of suicidal behavior. Furthermore, the finding of this association in subjects with a primary diagnosis of BPD highlights how the presence of subthreshold ATs may be a predictor of suicidal ideation or behaviors to be taken into close consideration, confirming the importance of investigating the presence of subthreshold ATs in clinical populations.

## Figures and Tables

**Table 1:** Age and gender comparison between diagnostic groups.

		Non Suicidal Mean ± SD, Mean Rank	Suic. Ideation Mean ± SD, Mean Rank	Suic. Behav. Mean ± SD, Mean Rank	H	*p*
Age		39.08 ± 12.60, 58.02	43.06 ± 17.03, 65.12	34.27 ± 13.39, 48.65	4.571	0.102
		n (%)	n (%)	n (%)	Chi-square	*p*
Gender	F	17 (68%)	9 (52.9%)	49 (76.6%)	3.742	0.154
	M	8 (32%)	8 (47.1%)	15 (23.4%)		

**Table 2:** Comparison of AdAS Spectrum scores among groups.

AdAS Spectrum Scores	Non-Suicidal Mean ± SD, Mean Rank	Suic. Ideation Mean ± SD, Mean Rank	Suic. Behav. Mean ± SD, Mean Rank	H	*p*
Child./adolesc.	7.76 ± 4.05, 37.16	9.47 ± 4.32, 48.88	11.42 ± 4.77, 61.11	11.415	0.003 *
Verb. comm.	6.00 ± 3.77, 35.44	7.47 ± 3.83, 49.47	8.92 ± 3.22, 61.63	13.510	0.001 *
Non-verb. comm.	9.08 ± 6.42, 30.58	13.11 ± 5.44, 55.35	14.97 ± 4.73, 61.96	18.899	0.000 °
Empathy	4.80 ± 2.94, 47.18	4.12 ± 2.31, 40.44	6.08 ± 2.93, 59.44	6.583	0.037 ^
Inflex. and routine	15.92 ± 7.68, 37.36	19.35 ± 7.69, 52.26	21.28 ± 8.14, 60.13	9.923	0.007 *
Restrict. interest and rum.	7.72 ± 4.63, 31.28	11.53 ± 3.06, 56.26	12.08 ± 3.93, 61.45	17.600	0.000 °
Hyper- hyporeact.	3.80 ± 2.90, 31.26	7.29 ± 3.23, 59.03	7.67 ± 4.26, 60.72	17.284	0.000 °
Total Score	55.08 ± 27.06, 30.44	72.35 ± 22.69, 50.59	82.42 ± 24.71, 63.28	20.706	0.000 °

* Suic. behav. > non-suicidal; ° suic. behav., suic. ideation > non-suicidal; ^ suic. behav. > suic. ideation.; significant for *p* < 0.05.

**Table 3:** Spearman’s correlations coefficients among AdAS Spectrum domains and total score and suicidal ideation and suicidal behaviors in the total sample. All correlations were statistically significant (*p* < 0.05).

	Suicidal Ideation	Suicidal Behaviors
Child./adolesc.	0.215 *	0.322 *
Verbal communication	0.282 *	0.383 **
Non-verbal comm.	0.421 **	0.376 **
Empathy	0.079	0.255 *
Inflex. and routine	0.279 *	0.289 *
Restrict. interest and rum.	0.360 **	0.340 **
Hyper- hyporeact.	0.335 **	0.364 **
AdAS Total Score	0.359 **	0.430 **

*: *p* < 0.05; **: *p* < 0.001; significant for *p* > 0.05.

**Table 4:** Logistic regression analysis with the presence of suicidal ideation as a dependent variable and AdAS Spectrum domains as independent variables, carried out on the whole sample.

		b (SE)	*p*	Exp (B)
Step 1	Constant	−1.356 (0.665)	0.041 *	0.258
	Restrict. interest and rum.	0.255 (0.067)	0.000 *	1.290
Cox & Snell R^2^ = 0.165; Nagelkerke R^2^ = 0.248; overall correctness percentage = 76.4%, * significant for *p* < 0.05				
Step 2	constant	−1.585 (0.699)	0.023 *	0.205
	Restrict. interest and rum.	0.171 (0.075)	0.022 *	1.187
	Hyper–hyporeact.	0.194 (0.089)	0.029 *	1.214
Cox & Snell R^2^ = 0.204; Nagelkerke R^2^ = 0.307; overall correctness percentage = 79.2%, * significant for *p* < 0.05				

**Table 5:** Logistic regression analysis with suicidal behaviors as a dependent variable and AdAS Spectrum domains as independent variables, in subjects with suicidal ideation only and suicidal behaviors.

		b (SE)	*p*	Exp (B)
Step 1	Constant	−0.043 (0.588)	0.942	0.958
	Empathy	0.271 (0.115)	0.018 *	1.312

Cox & Snell R^2^ = 0.079; Nagelkerke R^2^ = 0.123; overall correctness percentage = 80.2%; *: significant for *p* < 0.05.

## Data Availability

All data generated or analyzed during this study are included in this published article.
